# Disposable Fluorescence Optical pH Sensor for Near Neutral Solutions

**DOI:** 10.3390/s130100484

**Published:** 2012-12-28

**Authors:** Luca Ferrari, Luigi Rovati, Paola Fabbri, Francesco Pilati

**Affiliations:** Department of Engineering Enzo Ferrari, University of Modena and Reggio Emilia, via Vignolese 905, Modena I-41125, Italy; E-Mails: luigi.rovati@unimore.it (L.R.); paola.fabbri@unimore.it (P.F.); francesco.pilati@unimore.it (F.P.)

**Keywords:** optical sensor, pH sensor, fluorescence sensor, hydrogel, polymer matrix

## Abstract

The design, development and performance evaluation of a fluorescence-based pH sensor for on-line measurements is presented. The *pK_a_* of the sensing element has been calculated to be 7.9, thus the sensor is suitable for measurement of near neutral solutions. The sensor consists of a low-cost disposable polymer sensing probe, in contact with the solution under test, interrogated by an optoelectronic transduction system. The pH sensitive dye is based on fluorescein *O*-methacrylate, which has been covalently linked to a hydrogel matrix, realized through the use of HEMA (2-hydroxyethyl methacrylate), HDDA (1,6-hexanediol diacrylate) and PEGDA (polyethylene glycol diacrylate). The optical interrogation setup, together with the electronics, has been developed to acquire and process the fluorescence signal. The sensor works over a pH range between 6.5 and 9.0. In the range between 7.0 and 8.0, the sensor shows a linear behavior with a maximum linearity error of 5%. Thanks to the good performance of the sensing element and transduction system, the short term drift of the reading (measured over 40 min) is lower than 0.15%. The measuring system also exhibits good performance in terms of response time and reproducibility.

## 1. Introduction

pH (Latin: pondus hidrogenii) is a commonly measured parameter of great interest in many application fields, such as environmental monitoring [1,2], bioprocessing [3] and biomedical diagnostics [4]. The measurement of pH is routinely performed using the glass electrode. Nevertheless, the electrochemical approach suffers from many drawbacks, such as electromagnetic interference, difficulty in miniaturisation, and limitations when measuring aqueous suspensions of organic matter or low-ionic-strength solutions. With respect to electrochemical sensors, optical pH sensors allow for a higher sensitivity and selectivity, due to the luminescence phenomena. Moreover, they are insensitive to electromagnetic interference, they do not need any reference electrode and they are suitable for remote and disposable sensing, which is typical of in-situ applications [5,6].

Nowadays, the fluorescence phenomena is widely exploited to realize optical sensors, due to the higher sensitivity and versatility with respect to other detection schemes. In particular, fluorescein is widely used due to its high molar absorptivity at the wavelength of the argon laser (*λ* = 488 nm), high fluorescence quantum yield and pH-dependent emission spectra [7].

Several optical fluorescence-based pH sensors consist of a solid matrix permeable to protons, containing a pH sensitive dye, which can be reached by the analyte. Chemical bonding between the dye and the matrix is necessary to avoid undesirable leaching effects. pH measurements are performed revealing reversible variations in the intensity or lifetime of the fluorescent indicator; polymers are widely used as immobilization matrices for pH-sensitive fluorescent dyes, thanks to the possibility of molecular tailoring to control and tune proton absorption [8–12].

Nevertheless, although lots of pH sensors are commercially available at present, only few of them are suitable for the fabrication of disposable sensing elements [13]. In fact, the key aspect of a disposable sensor is a cheap and simple transduction mechanism, which converts the information of interest into a readable signal, so that it is possible to use the same optical and electronic instrumentation for different measurements changing only the cheap and disposable sensing element. Disposability is becoming a fundamental characteristic in life science sensors, where exams performed on different patients require always new and sterilized sensing elements in contact with biological tissues.

In this paper, we present a disposable fluorescence-based optical pH sensor for on-line measurements in near neutral solutions. In particular, in Section 2, the polymer sensing element, the optics and electronics are fully described. Section 3 deals with the characterization of the sensor and finally the results are discussed and conclusions are drawn in Section 4.

## 2. Materials and Methods

In the next sections, after a brief theoretical discussion, the developed measurement system is presented. It basically consists of three parts: (i) polymer sensing element in contact with the solution, (ii) optical reading head and (iii) front-end and signal elaboration electronics.

### 2.1. Theoretical Background

**Figure 1.**
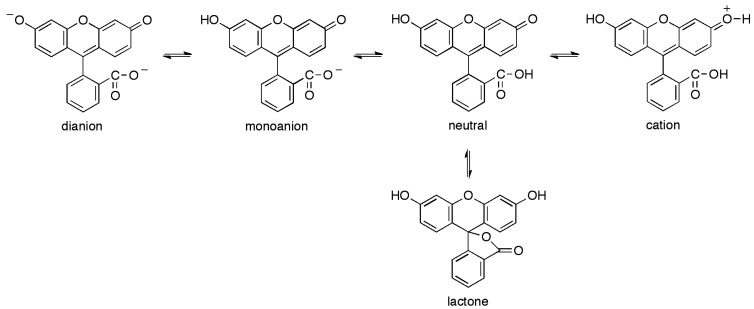
Ionization equilibria of fluorescein.

The development of the present sensor is based on the fluorometric determination of pH [14]. Following the absorption of a photon, the excited molecules can lose energy through non-radiative relaxation, emission of a photon or energy transfer to an acceptor. The reemitted photons usually possess less energy, so they are shifted to the red part of the spectrum. This property represents a great advantage, compared with the absorption spectroscopy, since it decreases the level of the shot noise, which is proportional to the square root of the light intensity. In fact, when the emission light is only observed, the signal-to-noise ratio is greatly improved. The sensor presented in this work makes use of a polymerizable fluorescein, namely fluorescein *O*-methacrylate, to detect pH changes in the measured fluorescence intensity. Fluorescein *O*-methacrylate is a new kind of pH-sensitive fluorescent monomer characterized by an excitation spectrum centered at *λ* = 490 nm and an emission spectrum centered at *λ* = 520 nm. Lots of optical pH sensors based on the dissociation equilibrium of fluorescent dyes are usually cross-sensitive to ionic strength. Nevertheless, fluorescein carries the least negative charges compared with other fluorescent indicators such as HPTS and carboxyfluorescein, which leads to a lower dependence on the ionic strength. Variations in ionic strength in the range from 50 mM to 400 mM causes a pH error of ca. 0.05 pH [15]. This error is acceptable and therefore fluorescein is a suitable indicator for pH measurement in aqueous solutions. Fluorescein exhibits multiple, pH dependent ionic equilibria [7]. Both the phenol and carboxylic acid functional groups of fluorescein are almost completely ionized in aqueous solutions above pH 9.0 (Figure 1). Acidification of the fluorescein dianion first protonates the phenol (*pK_a_* ≈ 6.4) to yield the fluorescein monoanion, then the carboxylic acid (*pK_a_* < 5.0) to produce the neutral species of fluorescein. Further acidification generates a fluorescein cation (*pK_a_* ≈ 2.1). Only the monoanion and dianion of fluorescein are fluorescent, with quantum yields of 0.37 and 0.93, respectively. A further equilibrium involves the formation of a colorless nonfluorescent lactone (Figure 1).

Nevertheless, the fluorescence emission spectrum of fluorescein, even in acidic solution, is dominated by the dianion, with only small contributions from the monoanion. Consequently, the wavelength and shape of the emission spectra resulting from excitation close to the dianion absorption peak at 490 nm are relatively independent of pH, but the fluorescence intensity is dramatically reduced under acidic conditions. The mass-action law relationships between pH and fluorescence intensity determines the response curve of this sensing approach [16]. Considering a dye covalently linked to a polymer matrix, defining *I_max_* and *I_min_* as the fluorescence contribution of the fully deprotonated and the fully protonated form of the fluorescent indicator, respectively, the relation between the fluorescence emission intensity *I_s_* and pH, in the presence of the sample under test, becomes [17–19]:

(1) $$pH=pK_{a}-b\cdot \text{log}\;\left(\frac{I_{\text{max}}-I_{s}}{I_{s}-I_{\text{min}}}\right)$$

where *pK_a_* is the acid-base constant of the indicator and b is a numerical coefficient, introduced to determine the slope of the function between *I_max_* and *I_min_*. In fact, the chemical and physical properties of the matrix including the dye (e.g., polarity and viscosity) could affect its sensitivity near the *pK_a_*, thus having different slopes for the same indicator in different matrices. Rewriting Equation (1) in terms of *I_s_* gives the well-known sigmoidal function [18]:

(2) $$I_{s}=\frac{I_{\text{max}}+I_{\text{min}}\cdot 10^{-(\frac{pH-pK_{a}}{b})}}{1+10^{-(\frac{pH-pK_{a}}{b})}}=I_{\text{min}}+\frac{I_{\text{max}}-I_{\text{min}}}{1+10^{-(\frac{pH-pK_{a}}{b})}}$$

This equation results in a nonlinear relationship between the fluorescence intensity *versus* pH, which has been used for the calculation of the *pK_a_* of the dye included in the polymer matrix.

### 2.2. Polymer Sensing Element

The polymer sensing element in contact with the solution transduces the level of pH into an optical information; it consists of a polymer matrix and a pH-sensitive dye covalently bonded to the polymer chains. The polymer matrix plays an important role because it is in direct contact with the solution and it contains the sensitive dye inside its molecular structure, so it has to be: (i) robust towards the flow, (ii) able to guarantee a fast penetration and mobility of the hydrogen ions, (iii) fast in response time, (iv) well adherent to the substrate of the measuring cell, (v) characterized by absence of or reduced dye leaching.

#### 2.2.1. Fabrication of the Polymer Matrix

**Figure 2.**
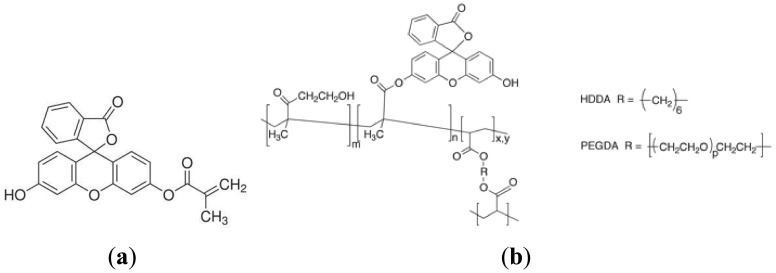
(**a**) Molecular structure of Fluorescein *O*-methacrylate. (**b**) Molecular structure of poly(HEMA) covalently bonded to Fluorescein *O*-methacrylate units.

**Figure 3.**
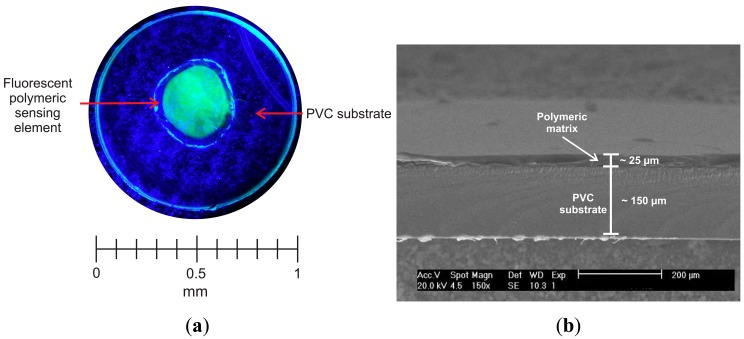
(**a**) Top-view picture of the hydrophilic polymer matrix including the fluorescent indicator, used as the sensing element, excited by a blue LED. (**b**) SEM cross-section picture of the hydrophilic polymer matrix including the fluorescent indicator, used as the sensing element.

All chemicals needed for the realization of a hydrophilic, highly swellable polymer matrix containing an optical sensing element were of analytical grade, purchased from Sigma-Aldrich (Milan, Italy) and used without further purification. All aqueous solutions were prepared using distilled water. The matrix was prepared by the free-radical polymerization of 2-hydroxyethyl methacrylate (HEMA), whose thermodynamic affinity for water is well known [20], catalysed by the radical photoinitiator 4-(2-hydroxyethoxy)phenyl-(2-hydroxy-2-propyl)ketone (Irgacure 2959, BASF, Ludwigshafen, Germany), used at 1.0% wt. The polymerization was run in the presence of a tetrafunctional monomer, namely 1,6-hexanediol diacrylate (HDDA) added at 5.0% wt with respect to HEMA, acting as crosslinker to avoid an excessive swelling degree of the acrylate matrix in contact with water, which could result in a poor mechanically resistance. Fluorescein O-methacrylate 97% (Figure 2(a)) was added to the acrylates mixture at 1.0% wt with respect to HEMA monomer; its methacrylate moiety allows for a covalent inclusion of the fluoresceine comonomer within the swellable polymer matrix, thus suppressing the severe drawback of indicator leaching. The covalent bonding between the HEMA matrix and the fluorescent dye is depicted in Figure 2(b). The overall swelling ratio of the polymer matrix in aqueous solutions was finely tuned by adding different amounts (up to 6.0% wt) of a flexible macromer, namely poly(ethylene glycol) diacrylate (PEGDA, Ebecryl13, Cytec Industries, Woodland Park, NJ, USA), to further improve the polymer chain flexibility, and in turn ion mobility within the polymer matrix [21]. The mixture of chemicals was accurately mixed using a magnetic stirrer before being deposited by spin coating (500 rpm, 30 s) onto a transparent PVC substrate (Figure 3(a)), and cured by UV-irradiation with a medium pressure mercury lamp under nitrogen with a light intensity on the surface of the sample of 30 *mW*/*cm*^2^ for different time up to 70 s.

Figure 3(b) reports a SEM micrograph of a cross-section of the sensing polymer film (thickness about 25 *μm*) deposited onto the PVC substrate (thickness 150 *μm*). Because of the manual realization of the sensing elements, it was difficult to firmly control the thickness of the polymer sensing layer; this disuniformity between different sensing elements may cause some variation in terms of response time and sensitivity. The polymer film uniformity could be improved through the application of instrumental deposition techniques, like the one reported by Tian *et al*. [22,23].

### 2.3. Optical Reading Head

**Figure 4.**
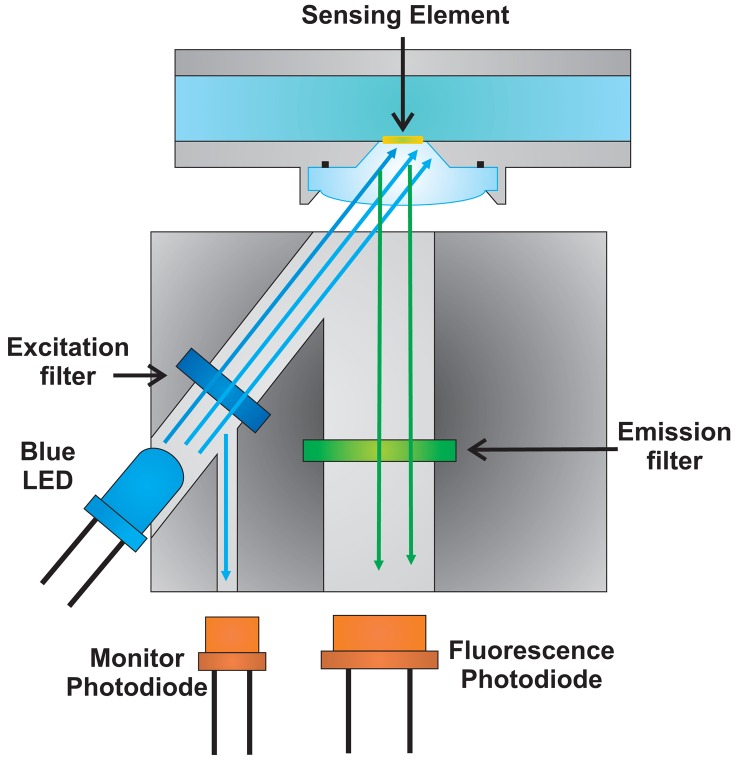
Schematic representation of the optical setup realized to perform fluorescence intensity measurements. It consists of a blue LED to excite the fluorescein, two optical filters (an excitation and an emission filter respectively) and finally two photodiodes, one to collect the fluorescence signal and one to monitor the optical power emitted by the LED.

A schematic representation of the realized optical setup is reported in Figure 4. To perform fluorescence intensity variation measurements, it is necessary to excite the fluorescein; in the present work excitation was induced by a blue LED (*λ_peak_* ≅ 480 nm), and the emitted fluorescence signal was collected. Since the optical power emitted by the LED is much higher than the emitted fluorescence, two filters inside the optical head have been positioned; in particular an excitation filter on the light emitted by the LED (*λ_cutoff_* = 485 nm, ODL S.r.l., BG12, Bergamo, Italy) and an emission filter (Δ*λ* = 515–535 nm, Thorlabs, MF525-39, Munich, Germany) on the optical path of the fluorescence light towards the photodetector. The unwanted excitation light collected by the fluorescence channel produces an offset in the signal of interest comparable with the offsets introduced by the electronics. Thus, it can be compensated by a proper calibration of the sensor. Finally, two photodiodes have been used, one to collect the fluorescence signal and one to monitor the optical power emitted by the LED. The monitor photodiode is used to detect possible variations of the excitation optical power.

### 2.4. Front-End and Signal Elaboration Electronics

**Figure 5.**
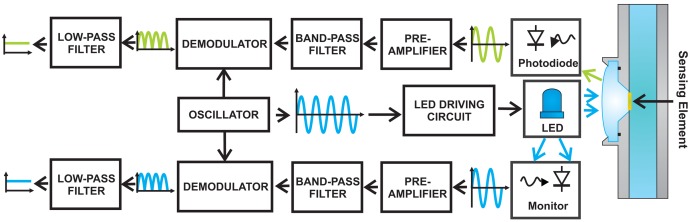
Schematic representation of the electronic board developed and realized to acquire and process the emitted fluorescence signal and the optical power emitted by the LED. The lock-in technique has been used, modulating the LED driving signal and demodulating the collected signal with the same sinusoidal signal. Only the signal of interest presents a non-zero average value.

An electronic board has been developed to drive the LED, acquire and process the emitted fluorescence signal and the optical power emitted by the LED. In order to reduce the effect of photobleaching, which is typical in fluorescent indicators, the blue LED was excited by current pulses (duration 800 ms) at a frequency of 0.125 Hz [24]. Moreover, to increase the robustness against the interferences and because of the low signal-to-noise ratio, the lock-in technique has been used, modulating sinusoidally the LED driving current during the pulse and demodulating the collected signal with the same signal (frequency = 6.06 kHz), as shown in Figure 5. In this way, only the signal of interest presents a non-zero average value, which is representative of the excitation and fluorescence intensities.

For the performed measurements, the fluorescence-to-monitor ratio was considered, thus avoiding that the variations in the optical power emitted by the LED could influence the fluorescence measurements. In fact, the fluorescence intensity *I_s_* is a function of the absorbed light [14], as reported in Equation (3):

(3) $$I_{s}=kI_{0}\phi \;{∊}_{\lambda }\;lC$$

where *I*_0_ is the intensity of the exciting beam, *ϕ* is the quantum yield of the fluorophore, ∊*_λ_* is the molar absorptivity at *λ_ex_*, *l* is the optical path length in the sample, *C* is the concentration of the fluorophore and *k* is an instrumental factor. Considering the fluorescence-to-monitor ratio, it yields:

(4) $$I_{\text{Ratio}}=\frac{I_{s}}{I_{0}}=k\phi \;{∊}_{\lambda }\;lC$$

As shown in Equation (4), the ratio signal is independent from the optical power emitted by the LED. In the next sections of the article, it will be referred to the fluorescence-to-monitor ratio as *I_Ratio_* and to the normalized fluorescence-to-monitor ratio as *I_Norm_*, defined as:

(5) $$I_{\text{Norm}}=\frac{{\text{I}}_{s}}{I_{0}\cdot I_{\text{Ratio}}{|}_{\text{max}}}$$

## 3. Experimental Results

**Figure 6.**
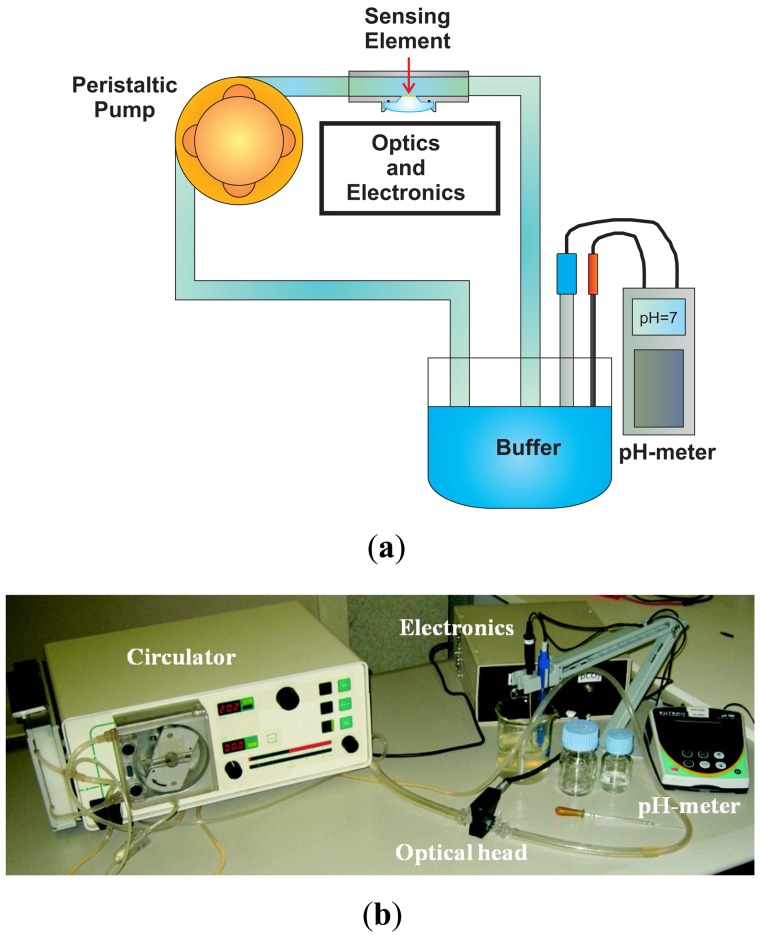
(**a**) Schematic representation of the fluidic system used to test the pH sensor. It consists of (i) a closed loop circulator, (ii) the optical head with the sensing element inside, (iii) a beaker with a reference glass electrode pH-meter to measure the pH of the solution and a thermocouple to measure the temperature of the solution and correct the pH value. (**b**) Picture of the fluidic system used to test the pH sensor.

In order to test the sensor, a fluidic system has been realized. As shown in the schematic representation in Figure 6(a) and in the picture in Figure 6(b), it consists of (i) a closed loop circulator, (ii) the optical head with the sensing element inside, (iii) a beaker with a reference glass electrode pH-meter (Eutech Instruments, XS pH 700, Nijkerk, The Netherlands) to measure the pH of the solution and a thermocouple to measure the temperature of the solution and correct the pH value.

All the measurements were performed at room temperature, with a flow rate of 200 mL/min. The pH of the solution was changed by adding acetic acid or ammonium hydroxide to the solution.

## 3.1. Characterization of the Sensor pK_a_

**Figure 7.**
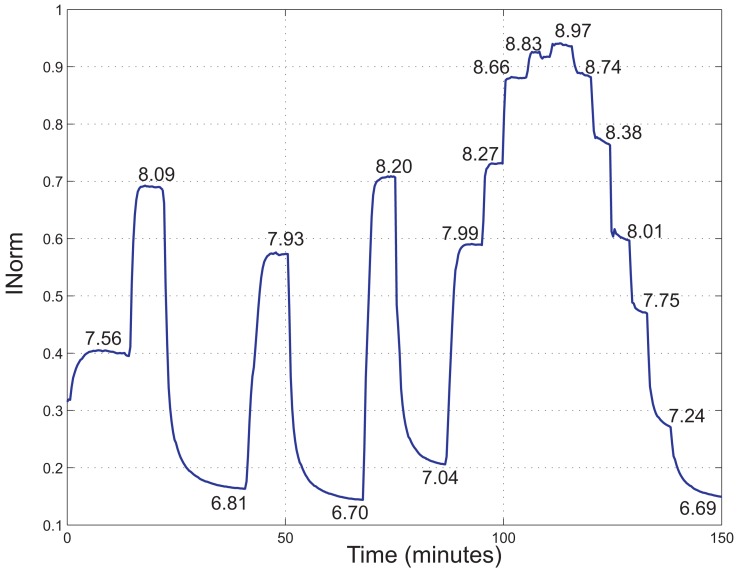
*I_Norm_ versus* time of a measurement performed in an aqueous solution, changing the pH value of the solution. The steady state pH values of the solution as measured by the reference instrument are reported close to the curve.

**Figure 8.**
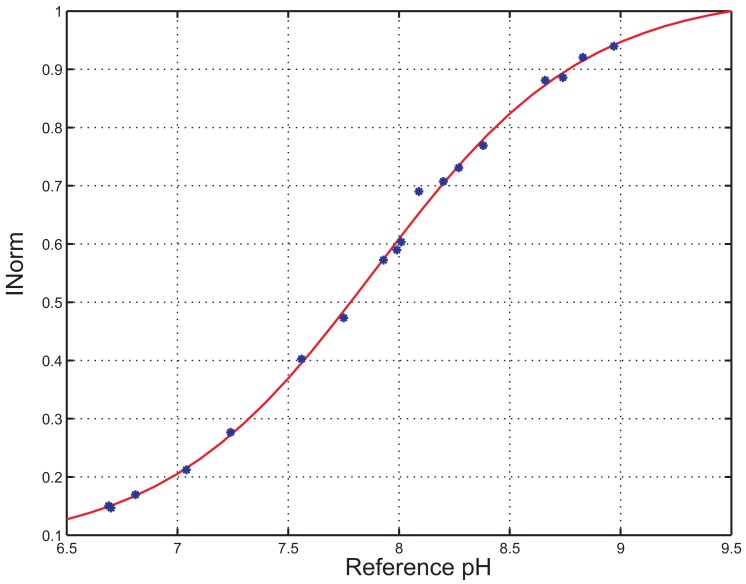
*I_Norm_ versus* reference pH and sigmoidal interpolation curve.

A fluorometric titration has been performed to investigate the *pK_a_* of the indicator inside the polymer matrix in aqueous solutions. In Figure 7, *I_Norm_ versus* time acquired at different pH values is shown. The steady state pH values of the solution as measured by the reference instrument are reported close to the curve. In Figure 8, the average of the *I_Norm_* signal over a time interval of 60 s for every reference pH value is shown.

*I_Norm_* values have been fitted to a sigmoidal function, represented in Figure 8 and determined by applying Equations (5) and (2), where *I_max_* = 1.98, *I_min_* = 1 and *b* = 1.10. The *pK_a_* of the indicator was thus estimated to be 7.9. This value is bigger than the one reported in literature for the fluorescein in aqueous solutions, *i.e*., 6.4 [25]. According to Vasylevska *et al.* [18], an increase *in pK_a_* is observed upon covalent immobilization of the indicator and is attributed to the decrease in the polarity of the microenvironment.

The sigmoidal interpolation curve can be approximated with a linear curve for a range of values of pH close to the *pK_a_* of the indicator. In particular, here we propose to consider a linearity range between 7 and 8. In the next section, the linearity characteristics of the sensor within this range are presented.

### 3.2. Evaluation of the Non-Linearity Error

**Figure 9.**
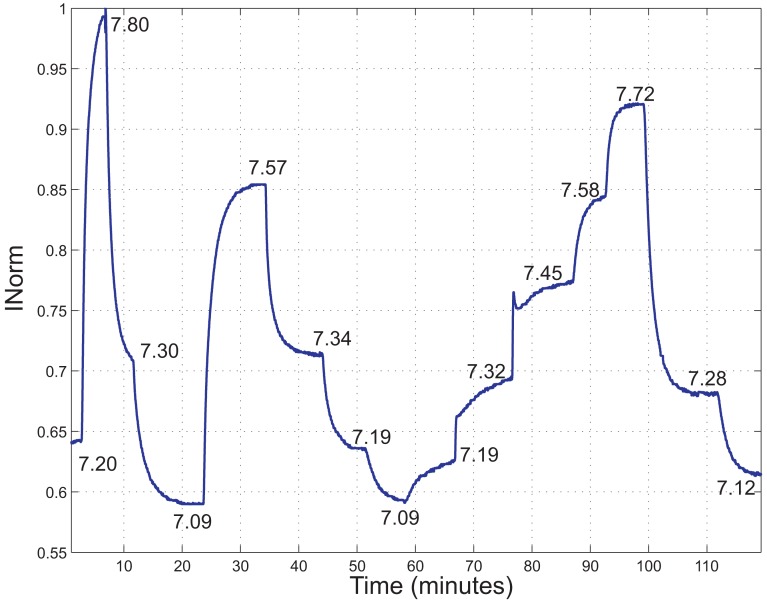
*I_Norm_ versus* time acquired at different pH values in the range 7.0–8.0. The steady state pH values of the solution as measured by the reference instrument are reported close to the curve.

In Figure 9, *I_Norm_ versus* time acquired at different pH values in the range 7.0–8.0 is shown. It can be noted that the new normalization of *I_Ratio_* tends to expand and shift the ranges of values of *I_Norm_* respect to the data shown in Figure 7.

For every reference pH value, the average of *I_Norm_* has been calculated over an interval of 60 s. Afterward, these values were fitted to a linear function as:

(6) $${\bar{I}}_{\text{Norm}}=0.546\cdot pH_{\text{Reference}}-3.285$$

where *Ī_Norm_* is the average value of *I_Norm_* and *pH_Reference_* is the pH measured by the reference pH electrode. Hence, the estimated pH values were calculated as:

(7) $$pH_{\text{Estimated}}=\frac{{\bar{I}}_{\text{Norm}}-q}{m}=\frac{{\bar{I}}_{\text{Norm}}+3.285}{0.546}$$

**Figure 10.**
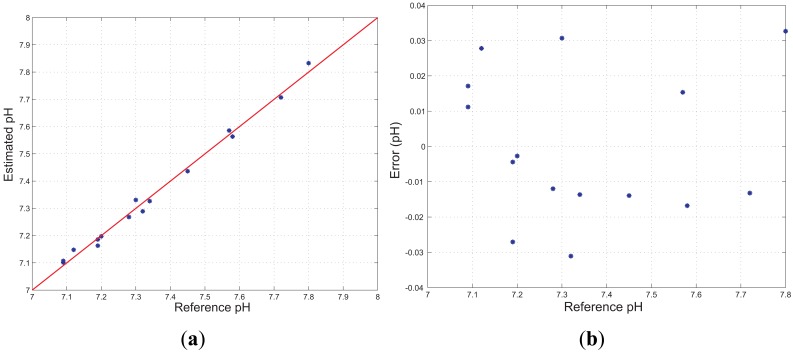
(**a**) Estimated *versus* reference pH and fitting line. (**b**) Difference between estimated and reference pH *versus* reference pH.

In Figure 10(a) the estimated values *versus* reference pH are shown, together with the fitting line (R = 0.9958), whereas Figure 10(b) shows the difference between the estimated and reference pH values, for every reference pH value. Thus, the Integral Non-linearity error, which is a parameter representing the maximum deviation between the sigmoidal interpolation curve of Equation (2) and the linear interpolation curve of Equation (6), was calculated to be 0.02 units of pH, which represents the 2% in the range 7.0–8.0. Finally, the Integral Non-linearity error was calculated also for the measurement reported in Figure 7 and was determined to be 0.05 units of pH, which represents the 5% in the range 7.0–8.0. These small variations between the Integral Non-linearity errors are probably due to different sensing elements used to perform these tests.

### 3.3. Short Term Stability

**Figure 11.**
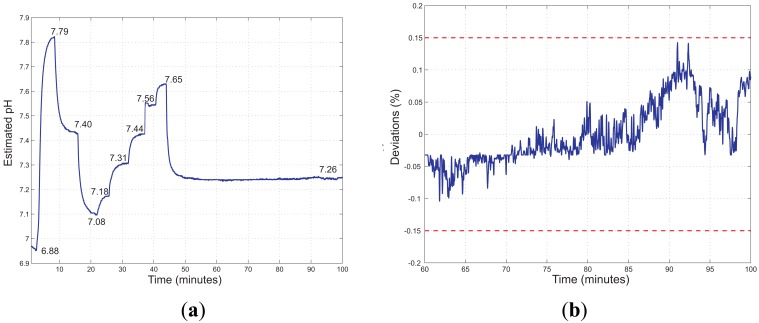
(**a**) Estimated pH *versus* time of a measurement performed changing the pH value of the solution and then keeping it constant. The steady state pH values of the solution as measured by the reference instrument are reported close to the curve. (**b**) Deviations of the estimated pH respect to its mean value in the time interval 60–100 min. All deviations are within 0.15% of the mean value of the estimated pH (dashed lines).

Figure 11(a) shows the estimated pH values, determined through Equation (7), *versus* time acquired firstly changing the pH of the solution and then keeping it constant. Assuming that the pH of the solution is completely stabilized at t = 60 min, the short term stability can be determined by evaluating the deviations of the estimated pH respect to its mean value in the time interval 60–100 min. As shown in Figure 11(b), no significant drifts have been observed in this time interval; all deviations are within 0.15% of the mean value of the estimated pH.

### 3.4. Response Time

**Figure 12.**
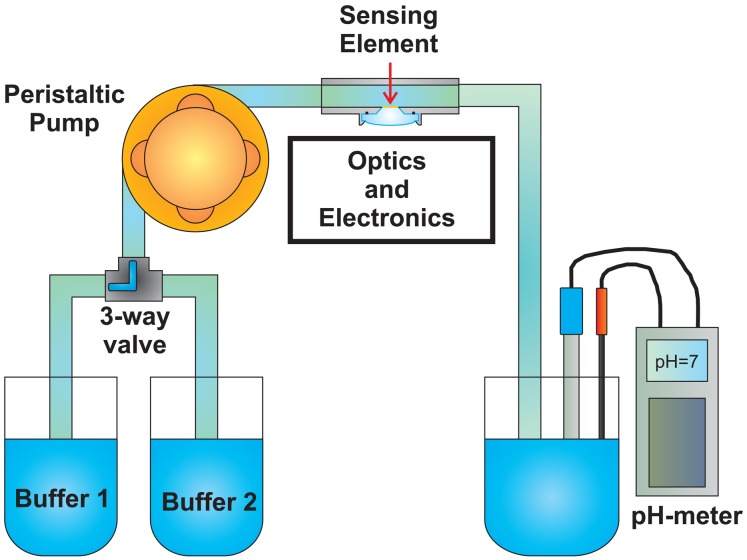
Schematic representation of the fluidic system used to measure the pH sensor response time. It consists of (i) a closed loop circulator, (ii) the optical head with the sensing element inside, (iii) a beaker with a glass electrode pH-meter to measure the pH of the solution and a thermocouple to measure the temperature of the solution and correct the pH value. A 3-way valve has been added to switch between two different buffer solutions.

The sensor response time, the so-called *τ*_90_, is defined as the time required for the sensor output to reach 90% of the change from its previous value to the final settled value. To measure the response time of the developed sensor, the fluidic system reported in Figure 12 has been realized.

**Figure 13.**
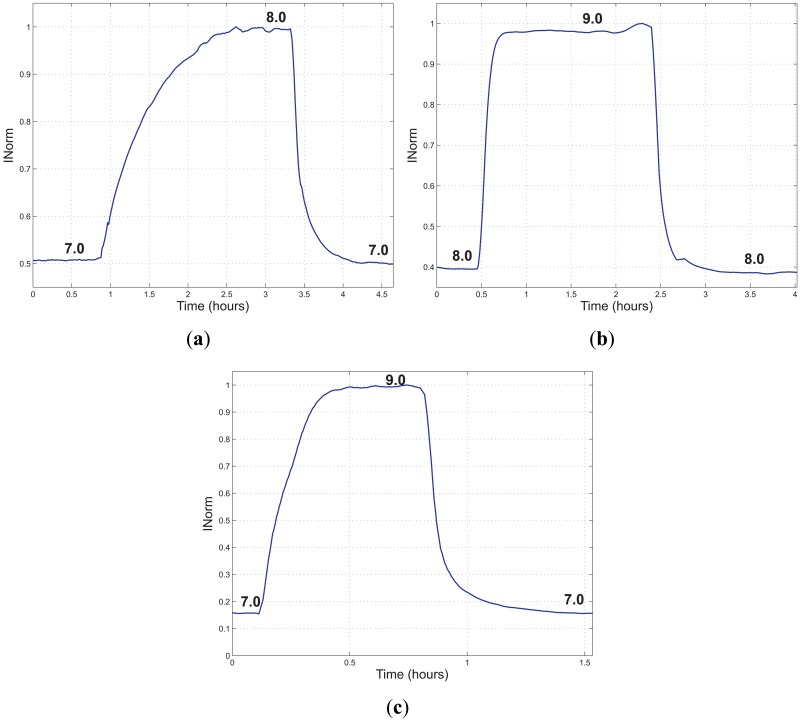
*I_Norm_ versus* time acquired during the three tests performed to evaluate the response time of the sensor and its dependency on the pH value. (**a**) Sensor response to a change in pH from 7 to 8 and back; (**b**) sensor response to a change in pH from 8 to 9 and back; (**c**) sensor response to a change in pH from 7 to 9 and back.

The setup shown in Figure 6(a) has been integrated by a 3-way valve, thus allowing switching between two different buffer solutions (Fisher Scientific, Sigma-Aldrich). The measurement was performed by pumping the first buffer solution till the sensor stabilized, then switching to the second buffer solution and waiting for the stabilization of the sensor and finally switching back to the first one. The measurements were performed with the same sensing element (50 *μm* thick), at room temperature at a flow rate of 200 mL/min. The whole circuit volume was about 200 mL. In Figure 13(a-c), *I_Norm_ versus* time is reported for three different test conditions.

In Table 1, the calculated rise and fall times (*τ*_90_) of the performed measurements are reported. The response time of the sensor depends on several factors, such as the flow-rate and the thickness of the film. Response times reported in Figure 13 are substantially different compared with those of Figures 7, 9 and 11(a). This large difference can be ascribed to the thickness of the sensing film. We have experimentally observed that even small differences in thickness can result in large changes in the dynamic response of the sensor. Moreover, the response time depends on the initial and final pH value. In fact, from the data reported in Table 1, it can be noticed that the response time is highly dependent on the pH values with respect to the *pK_a_* of the fluorescent dye. In fact, the response time is longer for the first variation of pH, (*pH* = 7.0 → 8.0, Figure 13(a)), which has been done between pH values below or equal to the *pK_a_* (*pK_a_* ≈ 7.9). If the final pH value is much higher than the *pK_a_*, the response time is significantly reduced; in fact, considering the second variation (*pH* = 8.0 → 9.0, Figure 13(b)), the response time is improved. Nevertheless, it can be noticed that, if the variation is done between two pH values chosen so that they are on either side of the *pK_a_* value, (*pH* = 7.0 → 9.0, Figure 13(c)), the rise and fall times are further improved (7 min per unit of pH and 13 min per unit of pH, respectively). Finally, it can be noticed that, if the final pH value is higher than the *pK_a_*, the rise time is lower than the fall time, whereas if the final pH value is lower than the *pK_a_*, the fall time is lower than the rise time.

Nevertheless, this dynamic behavior can be improved by using thinner polymer sensing elements. The thickness of the sensing element depends on a trade-off between the response time and the signal-to-noise ratio. As long as the signal-to-noise ratio of our sensor remains high enough, it is possible to use thinner polymer films and thus achieve shorter response time.

### 3.5. Reproducibility

**Figure 14.**
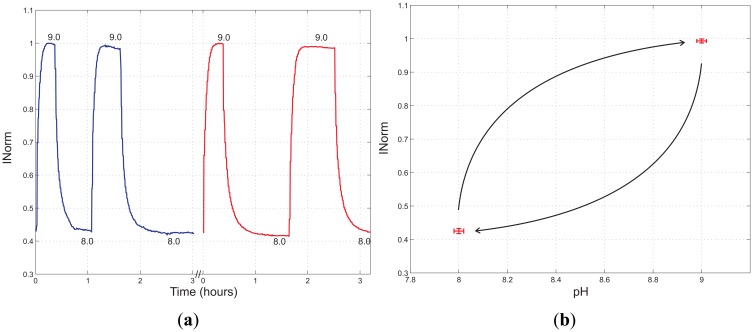
(**a**) Acquired *I_Norm_ versus* time changing the pH value between 8.0 and 9.0 to evaluate the reproducibility of the pH sensor. Two tests were performed (blue line and red line) on two consecutive days. (**b**) Mean values and error bars of the *I_Norm_* signal, measured at pH=8.0 and pH = 9.0.

The reproducibility of the pH sensor has been evaluated using the fluidic system represented in Figure 12, by changing the pH between the same two buffer solutions, *i.e*., 8.0 and 9.0 (Fisher, Sigma-Aldrich, Milan, Italy). In Figure 14(a), *I_Norm_ versus* time is reported. The first test (blue) was performed the day before the second test (red), using the same polymer sensing element. In Figure 14(b) the mean values of the *I_Norm_* signal, measured at *pH* = 8.0 and *pH* = 9.0, are reported, together with the error bars.

The mean values were calculated considering the average values of *I_Norm_* in the steady-states at pH = 8.0 and pH = 9.0 respectively. The error bars represent the standard deviations; for the reference pH values they were determined through the uncertainty reported on the datasheet of the buffer solutions, *i.e*., pH = 8.0 ± 0.02 and pH = 9.0 ± 0.02 respectively, whereas for the *I_Norm_* signal they were determined by the sample standard deviation (*s*). Finally, the mean values (*Ī_Norm_*) and the relative standard deviations (*σ* = *s*/*Ī_Norm_*) of *I_Norm_* were determined to be 0.425±1.80% at pH = 8.0 and 0.993±0.60% at pH = 9.0.

## 4. Discussion and Conclusions

A disposable optical fluorescence sensor has been developed. The sensor is based on the pH-dependent fluorescence of a purposely developed polymer matrix including a fluorescent monomer (fluorescein *O*-methacrylate 97%). This fluorescent monomer is covalently bonded to the hydrophilic polymer chains to realize a disposable sensing element without the risk of dye leaching. The optical head and the front-end electronics have been also developed to collect and process the fluorescence signal together with the electronics to acquire and process the signals of interest. Since the *pK_a_* of the sensing element has been calculated to be 7.9, the sensor is suitable for measurement of near neutral solutions. Good performance in terms of linearity (in the range 7.0–8.0), stability and reproducibility has been observed.

It has to be noticed that the response time strongly depends on the measuring conditions, *i.e*., amplitude of the pH range and its limit values. Obviously, this parameter also depends on the physico-chemical characteristics of the sensing element. In fact, it has been observed that the response time could change considerably for different sensing elements. This variation is mainly due to different thicknesses and cross-linking degrees of the sensing elements, caused by the manual realization. For a thickness of the sensing film of 50 *μm*, we measured a response time between 10 min and 1 h. For many applications, this dynamic response may be sufficient; nevertheless it is always possible to reduce the thickness of the polymer film to improve the response time. Furthermore, it was observed that the response time diverges in the case of pH excursions that have their final value close to *pK_a_* of the sensing element. Therefore, to improve the sensor dynamic response, this value can be tuned by adjusting the sensing matrix to prevent, in the application of interest, this condition to occur.

Future work will focus on the realization of the sensing elements in order to diminish the performance spread and improve their uniformity.

## Figures and Tables

**Table 1.** Rise and fall times estimated during the three performed tests.

	**Rise time***τ*_90_**(min)**	**Fall time***τ*_90_**(min)**
*pH* = 7.0 → 8.0	54.0	23.0
*pH* = 8.0 → 9.0	9.5	17.0
*pH* = 7.0 → 9.0	14.0	26.0

## References

[1] Dybko A., Wroblewski W., Rozniecka E., Pozniak K., Maciejewski J., Romaniuk R., Brzozka Z. (1998). Assessment of water quality based on multiparameter fiber optic probe. Sens. Actuators B Chem..

[2] Schirrmann M., Gebbers R., Kramer E., Seidel J. (2011). Soil pH mapping with an on-the-go sensor. Sensors.

[3] Kermis H.R., Kostov Y., Harms P., Rao G. (2002). Dual excitation ratiometric fluorescent pH sensor for noninvasive bioprocess monitoring: Development and application. Biotechnol. Progr..

[4] Gannot I., Ron I., Hekmat F., Chernomordik V., Gandjbakhche A. (2004). Functional optical detection based on pH dependent fluorescence lifetime. Lasers Surg. Med..

[5] Lin J. (2000). Recent development and applications of optical and fiber-optic pH sensors. Trends Anal. Chem..

[6] Bilro L., Alberto N., Pinto J.L., Nogueira R. (2012). Optical sensors based on plastic fibers. Sensors.

[7] Johnson I., Spence M.T.Z. (2010). Molecular Probes Handbook, A Guide to Fluorescent Probes and Labeling Technologies.

[8] Xu H., Sadik O.A. (2000). Design of a simple optical sensor for the detection of concentrated hydroxide ions in an unusual pH range. Analyst.

[9] Choi M.F. (1997). Spectroscopic behaviour of 8-hydroxy-1,3,6-pyrenetrisulphonate immobilized in ethyl cellulose. J. Photochem. Photobiol A Chem..

[10] Richter A., Paschew G., Klatt S., Lienig J., Arndt K.F., Adler H.J.P. (2008). Review on hydrogel-based pH sensors and microsensors. Sensors.

[11] Cajlakovic M., Lobnik A., Werner T. (2002). Stability of new optical pH sensing material based on cross-linked poly(vinyl alcohol) copolymer. Anal. Chim. Acta.

[12] Wencel D., MacCraith B., McDonagh C. (2009). High performance optical ratiometric sol-gel-based pH sensor. Sens. Actuators B Chem..

[13] Rovati L., Fabbri P., Ferrari L., Pilati F. (2011). Construction and evaluation of a disposable pH sensor based on a large core plastic optical fiber. Rev. Sci. Instrum..

[14] Kostov Y., Rao G. (2000). Low-cost optical instrumentation for biomedical measurements. Rev. Sci. Instrum..

[15] Weidgans B.M. (2004). New Fluorescent Optical pH Sensors with Minimal Effects of Ionic Strength. Ph. D. Thesis.

[16] Tusa J.K., Leiner M.J.P. Optodes Fluorescentes Pour Analytes de L'urgence.

[17] Povrozin Y.A., Markova L.I., Tatarets A.L., Sidorov V.I., Terpetschnig E.A., Patsenker L.D. (2009). Near-infrared, dual-ratiometric fluorescent label for measurement of pH. Anal. Biochem..

[18] Vasylevska A.S., Karasyov A.A., Borisov S.M., Krause C. (2007). Novel coumarin-based fluorescent pH indicators, probes and membranes covering a broad pH range. Anal. Bioanal. Chem..

[19] Szabelski M., Guzow K., Rzeska A., Malicka J., Przyborowska M., Wiczk W. (2002). Acidity of carboxyl group of tyrosine and its analogues and derivatives studied by steady-state fluorescence spectroscopy. J. Photochem. Photobiol. A Chem..

[20] Li L., Lee L.J. (2005). Photopolymerization of HEMA/DEGDMA hydrogels in solution. Polymer.

[21] Son Y.K., Jung Y.P., Kim J.H., Chung D.J. (2006). Preparation and properties of PEG-modified PHEMA hydrogel and the morphological effect. Macromol. Res..

[22] Tian Y., Su F., Weber W., Nandakumar V., Shumway B.R., Jin Y., Zhou X., Holl M.R., Johnson R.H., Meldrum D.R. (2010). A series of naphthalimide derivatives as intra and extracellular pH sensors. Biomaterials.

[23] Lu H., Jin Y., Tian Y., Zhang W., Holl M.R., Meldrum D.R. (2011). New ratiometric optical oxygen and pH dual sensors with three emission colors for measuring photosynthetic activity in cyanobacteria. J. Mater. Chem..

[24] Ferrari L., Fabbri P., Rovati L., Pilati F. Photobleaching Effects in Organic Thin Film Sensing Probes.

[25] Klonis N., Sawyer W.H. (1996). Spectral properties of the prototropic forms of fluorescein in aqueous solution. J. Fluorescence.

